# Broadband Dielectric Spectroscopy: A Viable Technique for Aging Assessment of Low-Voltage Cable Insulation Used in Nuclear Power Plants

**DOI:** 10.3390/polym13040494

**Published:** 2021-02-04

**Authors:** Davide Fabiani, Simone Vincenzo Suraci

**Affiliations:** 1 LIMES–Deptartment of Electrical, Electronic and Information Engineering–University of Bologna, 40136 Bologna, Italy; simone.suraci@unibo.it; 2 National Interuniversity Consortium of Materials Science and Technology (INSTM), 50121 Florence, Italy

**Keywords:** XLPE, dielectric spectroscopy, permittivity, radiation aging, oxidation, polymer degradation, post-irradiation effects

## Abstract

This paper deals with the study of a non-destructive technique to detect the aging state of cable insulation used in a nuclear environment subjected to radiation and temperature aging. Cable samples were aged under dose rates ranging from 0.42 and 1.06 kGy/h at 55 and 85 °C. The imaginary part of the permittivity at 100 kHz is found to correlate well with mechanical properties, such as elongation at break, which is typically used to diagnose cable insulation, but it is a destructive property and cannot be used on field. It has been demonstrated also that a post-irradiation effect occurs even years after aging is stopped, increasing the imaginary permittivity and worsening mechanical properties due to the slow conversion of radicals into oxidized species. The main consequence is that when cable insulation is subjected to a nuclear accident, releasing a huge amount of radiation, the health of cable insulation must be followed also for a long time after the accident occurred, since aging due to oxidation progresses even when the radiation source is switched off.

## 1. Introduction

Safety is the key feature of every component used in a nuclear power plant (NPP). In particular, critical elements have been designed in the past to last at least 40 years with an extremely high reliability. Since most of the NPPs were built in the 1970s or 1980s, such components are now reaching the end of life and they must be substituted or qualified again for another 20–40 years in a perspective of life extension of the existing NPPs. One of the key components, whose substitution has not been planned during service, is represented by low-voltage (LV) cables, which are used to deliver not only power but also instrumentation and control data, which is very important for operational safety. Since it has been estimated that more than 1500 km of LV cables are present in an NPP, the requalification of this huge number of elements can be a very critical issue for NPP life extension [1,2]. This requires the development of condition-based diagnostic techniques with the aim of assessing the degradation state of cable insulation and guaranteeing a sufficient residual life to withstand service and accidental conditions. Indeed, among the many requirements necessary to prolong the service life of existing cables, one of the most important regards the ability to withstand a loss of coolant accident (LOCA), which means that a cable must guarantee correct operation even after a severe nuclear accident.

To date, different condition monitoring (CM) techniques are available and, in some cases, standardized [3,4,5,6]. Many of those are laboratory-based condition monitoring techniques (e.g., elongation at break (EaB), Oxidation Induction Time (OIT), and density), which requires a small sample of the cable to be tested and destroyed. On the contrary, the most desirable feature for a CM technique is the possibility to be applied in situ in a non-destructive way. This would allow the state of the whole cable to be evaluated without the need of taking out a small length of it for laboratory analysis, which in some cases could be non-representative of the whole cable. Due to the huge number of cables to be qualified for each plant the use of non-destructive CM techniques is the only viable solution to save time and costs [7,8,9]. Among these techniques, the electric property measurement of cable insulation which can be obtained, e.g., through broadband dielectric spectroscopy (BDS), seems to provide promising results, since it allows an insulation degradation state to be evaluated in situ without damaging the cable [10,11,12,13,14,15,16,17].

As is known, environmental stresses, e.g., temperature and radiation, lead to the degradation of the cable and, in particular, of its electrical insulation by the action of oxygen, causing possible modifications of the dielectric response of the insulating material, which can be revealed by means of BDS. 

Among the environmental stresses, irradiation plays a major role leading to both physical and chemical degradation of the polymer. Irradiation of polymers leads to the creation of free active molecules (radicals) in different zones of the polymer (crystalline and amorphous phases), breaking bonds and creating new ones. 

Radiation yields to two main effects: crosslinking and oxidative degradation. The importance of one or the other of these phenomena is determined by the irradiation conditions and the structural features of the polymer. For XLPE, in particular, radiation is able to cause chain scission and free radical formation. In general, the higher the dose rate, the larger the number of radicals, and consequently, the greater the oxidation inside the polymer. On the other side, temperature stress has a double function: initiate the thermal–oxidative chain reactions (breaking R–H bonds and creating R°) and catalyze (or accelerate) the other oxidative reaction in the propagation step [18,19,20,21,22,23]. Therefore, the high-frequency dielectric response, as shown by previous works in the literature, could be related to the polarization of dipolar species (above all oxidized polymer chains). As a result of this, the high-frequency range of the imaginary part of permittivity could be linked to aging (oxidation) of the polymer [14,15].

Moreover, if radiation stress is particularly high, for example during a LOCA, oxygen in the air can be not enough to catalyze degradation reactions so that oxygen molecules can bond only on the outer layer of the insulation, preventing the oxidation from diffusing in the bulk. This effect is known in the literature as diffusion-limited oxidation (DLO). This phenomenon usually leads to a non-homogenous degradation throughout the insulation and to the creation of surface cracks, which can easily reduce the elongation at break (EaB) of these cables to values lower than 50%, which is considered typically as the limit value for cable requalification [24,25].

If a huge number of radicals remain trapped inside the crystalline phase, e.g., after an important irradiation absorption by the polymer, material properties can evolve even after the irradiation source is turned off. This phenomenon, called the post-irradiation effect, can affect the reliability of the cables qualification tests if they are conducted only immediately after a nuclear accident or LOCA [26,27]. 

This paper investigates the evolution of electrical properties of low-voltage cables used in NPP with thermo-radiative aging and the correlation with change in mechanical and chemical properties of the polymer. Moreover, the same cables have been tested again a long time later in order to evaluate possible post-irradiation effects and the long-term dielectric response of cables aged under different aging conditions.

## 2. Materials and Methods

### 2.1. Samples

**Figure 1:**
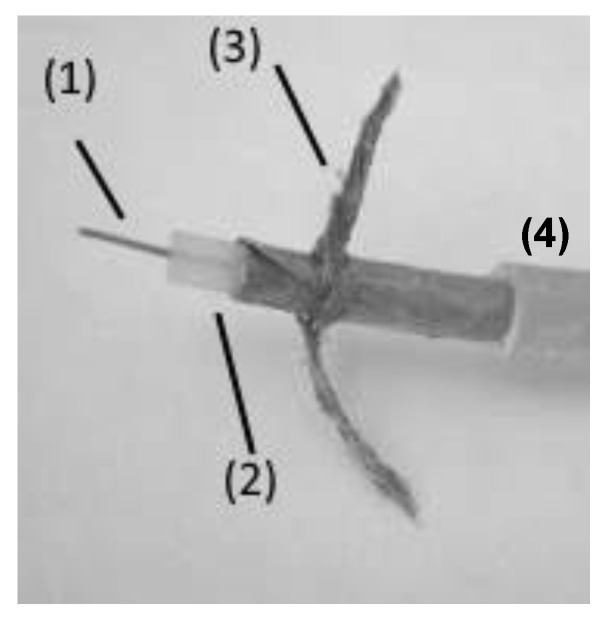
Morphology of the analyzed coaxial cables.

The samples investigated in this paper are low-voltage signal coaxial cables RG 59B (75 Ω of characteristic impedance, 400 V max operating voltage) manufactured by Alcatel, Lyon, France, which are commonly found in old NPPs, with insulation made by crosslinked polyethylene (XLPE). The structure of the analyzed cables is reported in Figure 1. Specimens are made of four concentric parts:

1. Conductor: Copper, diameter = 0.6 mm^2^ (the innermost);
2. Primary insulation: XLPE + additives (antioxidants + fire retardants), thickness 1.6 mm;
3. Shielding: Copper wire braid;
4. Outer sheath: PVC.

The outer sheath has not been considered in this paper, since it is not part of the main cable insulation.

### 2.2. Accelerated Aging

Aging has been performed in the Roza facility at UJV, Rez, Czech Republic. A ^60^Co γ-ray source was used to fulfill the process. Three different dose rates were applied, i.e., 0.42, 0.76, and 1.06 kGy/h for 1000 h at 85 and 55 °C in order to compare how different absorbed doses and temperatures can affect the physical and chemical properties of cables. Five sampling were carried out at time intervals of 200 h. 

### 2.3. Experimental Measurements

Electrical properties, in particular the complex permittivity, are detected by means of the broadband dielectric spectroscopy (BDS) technique through a Novocontrol Alpha Dielectric analyzer (Montabaur, Germany).

The complex permittivity is described by the following equation [11]:

(1) $$\dot{\varepsilon }=\varepsilon ’-j\varepsilon ”$$

where *ε*’ is the real part of permittivity defined as the dielectric constant of the material; *ε*” is the imaginary part of permittivity related to the dielectric losses of the material.

The instrumentation has been set with the following test parameters:

- Applied voltage: 3 Vrms;
- Frequency range: 10^−1^–10^6^ Hz;
- Room temperature.

Voltage is applied to the cable under test, 40 cm long, by connecting both ends of the inner conductor to one terminal of the BDS device through a BNC connector, while the external shield of the cable is connected to the return terminal of the device, so that the voltage is applied across cable insulation.

The device requires, as an input, the reference capacitance in vacuum, which is evaluated reducing the cable geometry to a plane capacitor. The reference capacitance is calculated through:

(2) $$C_{0}=\varepsilon_{0}\frac{\pi {\left(\frac{D_{eq}}{2}\right)}^{2}}{d}$$

where *ε*_0_ is the permittivity in vacuum; *D_eq_* is the equivalent plane diameter; and *d* is the thickness of the insulation.

Equivalent diameter is calculated through the following equation [28]:

(3) Deq=8dLln(R2R1)

where *L* is the length of the metallic mesh; and *R*_1_ and *R*_2_ are the inner and outer radius of the electrical insulation, respectively.

Cables were tested immediately after aging, 1 year later and after five years of shelf life, during which they were stored in uncontrolled air conditions, in order to investigate possible post-irradiation effects.

Elongation at break (EaB) was measured as an average of a sample of five cable specimens using a Universal testing machine by Instron, Norwood, MA, USA, with a load cell of 100 N. Tubular specimens, created by removing the external metallic shield and the internal copper wire with a length of 50 mm, were tested with a stress ramp speed of 20 mm/min.

Fourier Transform Infrared (FTIR) spectra have been recorded through a PerkinElmer Frontier spectrometer, Weltham, MA, USA, equipped with a diamond/ZnSe crystal in attenuated total reflectance (ATR) mode. Each spectrum is given by the average of 16 scans in the spectral range from 4000 to 650 cm^−1^, with a resolution of 4 cm^−1^. In order to investigate the oxidation development occurring inside the polymer during aging, the carbonyl index (CI) is calculated as the ratio between the area of the carbonyl band at 1720 cm^−1^ and the area of the methylene band at 2850 cm^−1^, whose value is not supposed to change during aging (reference peak).

## 3. Results

### 3.1. Tests Made Immediately after Aging

**Figure 2:**
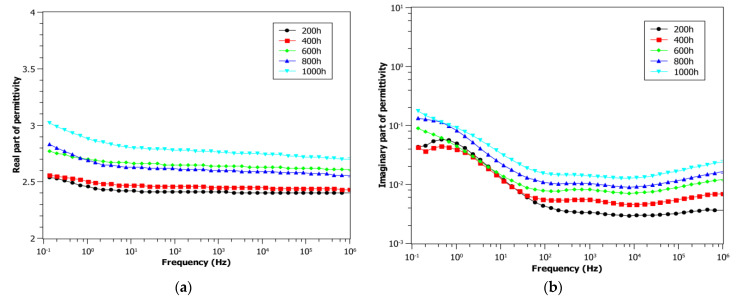
Real (**a**) and imaginary (**b**) part of the permittivity for cables aged at 85 °C and 0.42 kGy/h as a function of aging time.

Figure 2 shows BDS results made on cables aged at 0.42 kGy/h and 85 °C, immediately after the end of the respective aging times. In particular, the real part, *ε*’, and imaginary part, *ε*”, of permittivity are reported in Figure 2a,b. It can be clearly observed that both the real part and imaginary part increase with aging time in the whole frequency range: even if the *ε*’ rise with aging is quite small, about 20%, the variation of *ε*” at 0.1 Hz and, particularly, at 100 kHz is much larger, i.e., around one order of magnitude, while the effect is more limited in the range 1–100 Hz.

**Figure 3:**
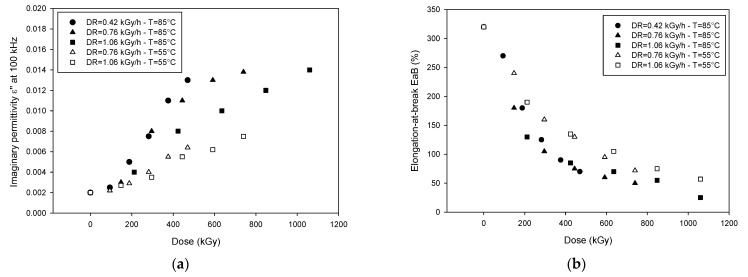
Imaginary part of the permittivity at 100 kHz (**a**) and elongation at break, EaB, (**b**) as a function of total absorbed dose for different combinations of dose rates and temperatures (after [15]).

Previous works [13,14,15,16] have shown that generally high-frequency imaginary permittivity, being due to mainly dipolar polarization, may be associated with oxidation products, generated by thermo-radiative aging, which typically exhibit a highly dipolar behavior. For this reason, the imaginary permittivity at 100 kHz has been extracted from experimental data for further investigations. Figure 3 displays *ε*’’ at 100 kHz (Figure 3a) and elongation at break (Figure 3b) for samples aged at different dose rates from 0.42 to 1.06 kGy/h and temperatures (55 and 85 °C) as a function of total absorbed dose (dose rate × aging time).

As can be seen from Figure 3a, imaginary permittivity increases with the increasing of dose, as expected, but the different aging conditions deeply influence the dielectric response of the material. In particular, at 85 °C and at high dose rate (1.06 kGy/h), a smaller variation of *ε*” at the same dose can be observed, with respect to lower dose rates. At lower temperature, on the contrary, the values of *ε*” depend only on the total dose and not on the dose rate. Figure 3b shows that EaB decreases with the increase of the absorbed dose, but a steep reduction is observed at higher dose rates and temperatures. 

This phenomenon is imputable to the diffusion-limited oxidation (DLO). In fact, high dose rates and temperature may lead to a non-homogenous oxidation of the polymer. Radiation and temperature catalyze the oxidative reactions, which require oxygen from the surrounding environment. If the amount of oxygen is not enough to bond with radicals made by high radiation dose rates, oxygen molecules cannot migrate to the inner part of the insulation and remain trapped in the outermost layers, causing DLO. Macroscopically, this behavior results in a more aged external layer of the cable, which can yield to the possible formation of cracks on the insulation surface, thus significantly reducing the values of EaB. 

**Figure 4:**
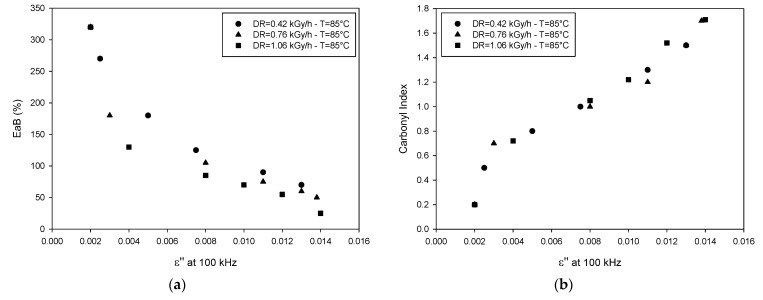
Cross-correlation plots: EaB vs. imaginary permittivity (**a**) and carbonyl index vs. imaginary permittivity (**b**) (after [15]).

The monotone behavior of EaB and *ε*” at 100 kHz with dose suggests a possible correlation among the two quantities, which is clearly highlighted in Figure 4a. The correlation is quite good, indeed, except for some points relevant to the high dose rate, which, as said above, are affected by DLO. If the limit point of EaB is taken at 50%, as suggested by the International Atomic Energy Agency (IAEA) recommendation [1], a value of *ε*” about 0.014 can be considered as the end point to assess the insulation capability for this specific cable.

It is worth noting that the increase of *ε*”, and consequent reduction of EaB, is associated with polymer oxidation, as confirmed by the rise of carbonyl index with aging time measured by FTIR, which is very well correlated with the rise of *ε*”, see Figure 4b.

### 3.2. Post-Irradiation Results

**Figure 5:**
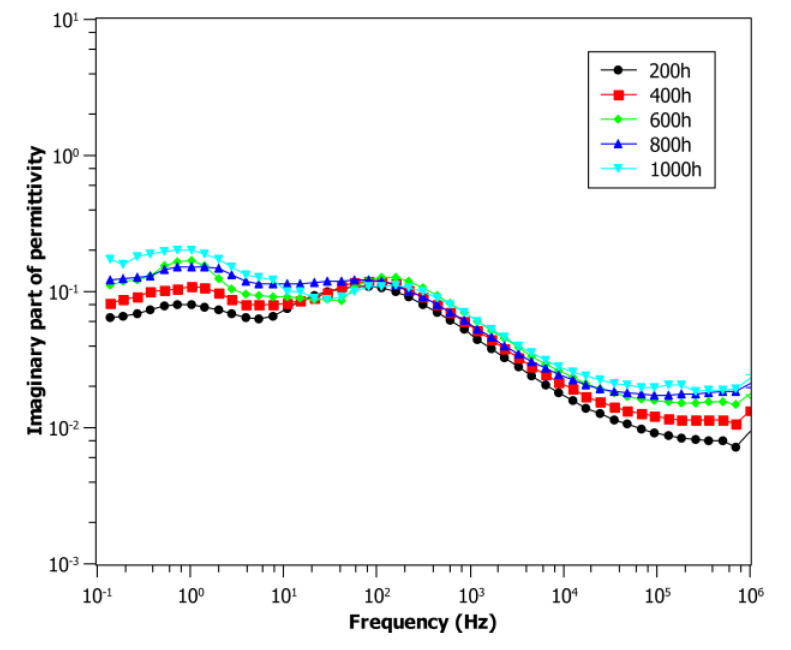
Dielectric spectra of imaginary part of permittivity at different aging times at dose rate 0.42 kGy/h and 55 °C. Tests made at after 5 years of storage.

**Figure 6:**
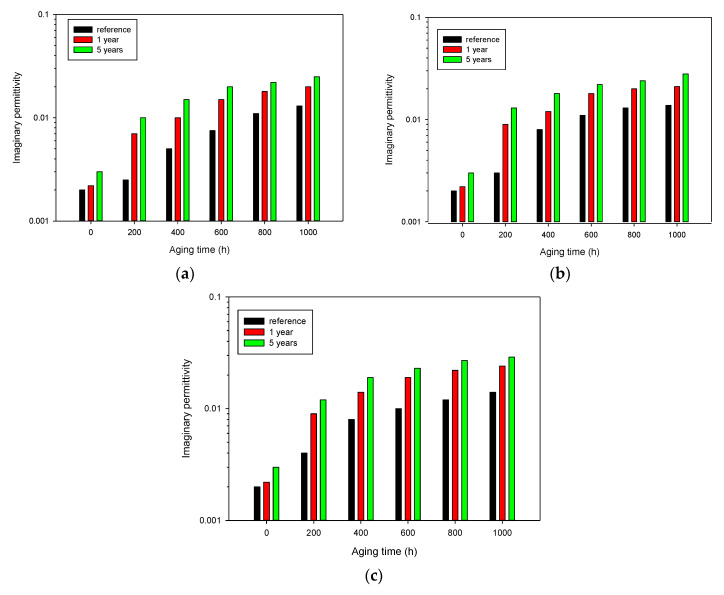
Imaginary permittivity at 100 kHz as a function of aging time at 0.42 kGy/h (a), 0.76 kGy/h (b), and 1.06 kGy/h (c), 85 °C, measured immediately after aging (reference), after 1 year and 5 years of storage in ambient conditions.

Figure 5 shows the trend of the imaginary permittivity for the same cables aged at 0.42 kGy/h and 85 °C, whose spectra are reported in Figure 2, after five years of storage in an uncontrolled environment (at room temperature and exposed to environmental moisture). Comparing Figure 2 (reference) and Figure 5 (storage time 5 years), one can observe that the dielectric response significantly changes over time due to the so-called post-irradiation effects. This latter can be explained considering the different diffusion rate of oxygen throughout the crystalline and amorphous phase. Indeed, diffusion constants for crystalline regions may be eight to nine orders of magnitude smaller than in the amorphous regions [26]. As mentioned before, the irradiation of polymers causes the formation of radicals in different areas of the polymer (amorphous and crystalline phases). However, while radicals in the amorphous region are able to bond to each other or with oxygen, radicals created in the crystalline region remain trapped for a certain period of time after irradiation. These trapped radicals can react inside the crystalline area, changing the properties of the material. Moreover, these radicals slowly migrate to the interface between the crystalline and amorphous region, where they are free to bond with the environmental oxygen, leading to further oxidative degradation, even a long time after the irradiation source has been turned off [26,27]. This phenomenon can be clearly noticed due to the change of the electrical response of all the aged cable samples after a given storage time, see Figure 6, where the imaginary permittivity at 100 kHz has been reported for cable samples aged from 0 to 1000 h at 0.42 kGy/h (Figure 6a), 0.76 kGy/h (Figure 6b), 1.06 kGy/h (Figure 6c), as received, after 1 year and 5 years of storage. The imaginary permittivity significantly increases for all the aging conditions during storage time, particularly in the first year, but also after 5 years, the property still varies, suggesting that cables were subjected to very long post-irradiation effects.

In general, the trend is quite similar for the three different aging conditions, i.e., the largest variation of imaginary permittivity occurs on the samples aged for 200 and 400 h. This behavior suggests that even if the total absorbed dose is very different for each considered aging conditions and for the various aging times, the radical amount created by radiation in the crystalline region can saturate soon so that the excess of absorbed dose can be less effective. Such reactive species may experience oxidative reactions in much longer times, even years after the aging source is turned off. Therefore, the polymer subjected to different environmental stresses may result in a similar degradation state and, consequently, a similar dielectric response after a long time. This situation is quite unusual, since post-irradiation effects have been observed on pure polymers only within 6–9 months after aging is stopped [26]. The longer times here observed could be explained considering that we are testing real cable insulation, where additives, particularly fire-retardants, can reach 50/60% of the insulation weight, hindering further radical and oxygen diffusion, thus slowing down oxidation reactions.

**Figure 7:**
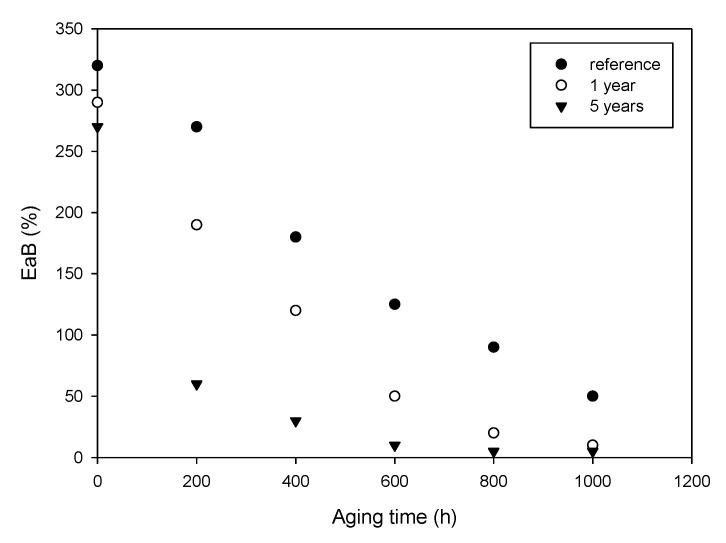
EaB of post-irradiated cable aged at 85 °C and 0.42 kGy/h measured immediately after aging (reference), after 1 year and 5 years.

The measurements of EaB performed on the same cable samples confirm that aging continued during the storage time, see Figure 7, which shows the trend of EaB for cables aged at 85 °C and 0.42 kGy/h. Tensile tests have been performed at delivery time, after one year and five years of storage. As expected, mechanical properties are dramatically affected by post-irradiation phenomena, which continue to alter the response even after some years. As an example, at delivery time, 1000 h of aging are needed to obtain an EaB value of ≈50%, after one year, the aging period is reduced to 400 h and, eventually, after 5 years of storage, even the 200 h-aged sample has an EaB lower than 50%.

**Figure 8:**
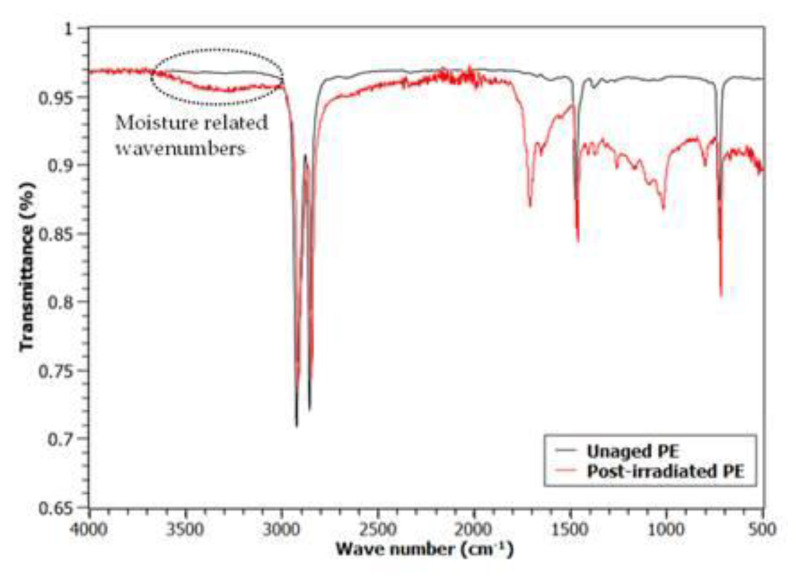
Example of ATR FTIR spectrum of a post-irradiated sample with reference to the unaged one.

In addition to post-irradiation effect, another phenomenon that must be taken into account during storage in an uncontrolled environment is moisture absorption by the primary insulation, particularly if this latter contains hygroscopic additives, such as, e.g., fire retardants. This can affect the low-frequency region of permittivity increasing both *ε*’ and *ε*” with the lowering of the frequency, sometimes with the appearance of a peak between 10 and 1000 Hz, as can be seen comparing Figure 2 and Figure 5 [29]. This behavior is observable in all the tests made after storage, and it can be related to moisture absorption that occurred during the shelf life, as confirmed by the FTIR spectra reported in Figure 8, which shows a non-negligible presence of moisture in post-irradiated samples. This is also a consequence of oxidation products, which build up during storage time and thanks to their polarity attract water molecules, as observed by Pourmand et al. on irradiated samples of Ethylene-Propylene Diene Monomer (EPDM) [23].

## 4. Conclusions

In conclusion, it has been shown that dielectric spectroscopy, particularly imaginary permittivity at high frequency, is well correlated with thermo-radiative aging of low voltage cable insulation This technique can be used for diagnostic or qualification purposes, provided that oxidation is the dominant degradation mechanisms, which can occur especially when antioxidants lose their efficiency during aging. Moreover, this method is non-destructive and can be applied to the whole cable length from the terminations, thus allowing a fast checkup of several cable assets.

Another important issue is relevant to post-irradiation effects which cause the degradation of the cable insulation also after radiation aging is stopped. This can be observed by looking at the dielectric permittivity values which continue to increase with time, with respect to those obtained immediately after aging. Moreover, different initial aging conditions can result in similar dielectric and mechanical properties years after the irradiation, reaching a sort of saturation. This effect can be very important in cable qualification after nuclear accidents, where high dose rates can impact on the primary insulation, and cable health condition is commonly evaluated immediately after the event, while polymer degradation can evolve even years after the accident has occurred. Therefore, under these circumstances, the assessment of cable insulation cannot be performed only once, but it should be repeated also a long time after the accident has occurred. Again, broadband dielectric spectroscopy can help in diagnosing cables with significant reliability issues.

## Data Availability

Not applicable.
